# Microfluidic-Based Non-Invasive Wearable Biosensors for Real-Time Monitoring of Sweat Biomarkers

**DOI:** 10.3390/bios14010029

**Published:** 2024-01-04

**Authors:** Seyedeh Rojin Shariati Pour, Donato Calabria, Afsaneh Emamiamin, Elisa Lazzarini, Andrea Pace, Massimo Guardigli, Martina Zangheri, Mara Mirasoli

**Affiliations:** 1 Department of Chemistry “Giacomo Ciamician”, Alma Mater Studiorum, University of Bologna, Tecnopolo di Rimini, Via Dario Campana 71, I-47922 Rimini, Italy; seyedeh.shariatipou3@unibo.it (S.R.S.P.); afsaneh.emamiamin@studio.unibo.it (A.E.); 2 Department of Chemistry “Giacomo Ciamician”, Alma Mater Studiorum, University of Bologna, Via Francesco Selmi 2, I-40126 Bologna, Italy; donato.calabria2@unibo.it (D.C.); elisa.lazzarini6@unibo.it (E.L.); andrea.pace7@unibo.it (A.P.); massimo.guardigli@unibo.it (M.G.); 3 Interdepartmental Centre for Industrial Aerospace Research (CIRI AEROSPACE), Alma Mater Studiorum, University of Bologna, Via Baldassarre Canaccini 12, I-47121 Forlì, Italy; 4 Interdepartmental Centre for Industrial Research in Renewable Resources, Environment, Sea, and Energy (CIRI FRAME), Alma Mater Studiorum, University of Bologna, Via Sant’Alberto 163, I-48123 Ravenna, Italy; 5 Interdepartmental Centre for Industrial Agrofood Research (CIRI AGRO), Alma Mater Studiorum—University of Bologna, Via Quinto Bucci 336, I-47521 Cesena, Italy; 6 Interdepartmental Centre for Industrial Research in Advanced Mechanical Engineering Applications and Materials Technology (CIRI MAM), Alma Mater Studiorum, University of Bologna, Viale Risorgimento 2, I-40136 Bologna, Italy

**Keywords:** wearable biosensors, point-of-care, sweat, colorimetric assay, electrochemical assay, enzymatic assay, immunoassay

## Abstract

Wearable biosensors are attracting great interest thanks to their high potential for providing clinical-diagnostic information in real time, exploiting non-invasive sampling of biofluids. In this context, sweat has been demonstrated to contain physiologically relevant biomarkers, even if it has not been exhaustively exploited till now. This biofluid has started to gain attention thanks to the applications offered by wearable biosensors, as it is easily collectable and can be used for continuous monitoring of some parameters. Several studies have reported electrochemical and optical biosensing strategies integrated with flexible, biocompatible, and innovative materials as platforms for biospecific recognition reactions. Furthermore, sampling systems as well as the transport of fluids by microfluidics have been implemented into portable and compact biosensors to improve the wearability of the overall analytical device. In this review, we report and discuss recent pioneering works about the development of sweat sensing technologies, focusing on opportunities and open issues that can be decisive for their applications in routine-personalized healthcare practices.

## 1. Introduction

Wearable biosensors have gained great attention in the last few years thanks to their potential to provide real-time information about performance and health status [1,2,3,4,5,6]. The possibility of developing wearable sensors exploiting flexible and comfortable clothing, patches, tattoos, or wristbands will allow for the monitoring of health parameters without expensive and bulky equipment. By integrating this approach with big data elaboration processes, it will also be possible to generate warnings about health-related problems that will be diagnosed at early stages, thus paving the way for a real preventive healthcare strategy.

The first proposals in this area reported physical sensors for monitoring steps, calories burned, or heart rate. With the improvement in technologies and the development of point-of-care (POC) devices, wearable biosensors started to change in order to fulfill the need for a sensing platform suitable for measuring health-related parameters. The analysis of biomarkers indicative of health disorders and diseases can be obtained by coupling sensing biological recognition elements (i.e., enzymes, antibodies, DNA-based probes, etc.) with a wearable, flexible platform suitable for performing a sensitive bioassay. The development of such biosensing devices should take into consideration several issues.

a. Sample to be analyzed. One of the most challenging aspects is the choice of biofluid to be analyzed. For wearable applications, sweat seems to be the ideal sample since it is easily and rapidly obtainable as it is produced by sweat glands that are distributed across the entire body (more than 100 glands/cm^2^ of skin) [7]. This biofluid had never been taken into great consideration for diagnostic purposes, but the recent interest in non-invasive sampling has highlighted its potentiality as it contains metabolites (i.e., lactate, glucose, urea, ethanol, or cortisol), electrolytes (i.e., sodium, potassium, chloride, or ammonium), trace elements (i.e., zinc or copper), and biomarkers (i.e., proteins, hormones, nucleic acids, neuropeptides, or cytokines) even if at low concentrations [8]. Despite the great number of studies involving sweat, there is still a lack of validation of the clinical value of several biomarkers. Indeed, a reliable correlation of the concentration of target analytes between blood and sweat levels is difficult since they are transported into sweat from capillaries with unique partitioning profiles. Furthermore, analytes can reach the sweat by passive (i.e., diffusion) or active mechanisms and can also be generated within the sweat duct itself [7]. 

b. Sampling and microfluidics. Non-invasive sampling at skin levels allows for the avoidance of issues related to blood sampling while obtaining information about physiological health status such as hydration and physical stress, as well as diabetes or cystic fibrosis. Even if sweat can be collected easily and rapidly without an invasive procedure, quantitative analysis of sweat still remains an open challenge since sweat is subjected to liquid evaporation, low and/or irregular volumes, contaminations, and interferences from the environment, as well as the fact that its contents (in terms of electrolytes and metabolites) change at excretion time [9,10,11]. A suitable sampling approach is then crucial to performing an effective and reproducible analysis, and this can be achieved by designing proper microfluidics [12,13]. Generally, most of the reported work relies on the use of an adsorbent adhesive pad able to store sweat, which is then analyzed using laboratory equipment. Researchers tried to fill this gap by exploiting microfluidic patterns for storing a precise volume of sweat and enabling them to perform the analysis at the same time to avoid contamination and evaporation, which can alter the concentration of the target analyte [14,15,16,17,18,19]. Microfluidics should provide continuous sampling, sending sweat through a fluidic channel into a measurement chamber [20], and it should also comprise reservoirs for reagent storage. Several designs were tested, including chrono-sampling devices for sweat collection based on a valving system, which enabled the analysis of the precise volume of sweat samples that are stored in reservoirs with a defined volume at a specific time [21,22]. Lin et al. employed hydrogel valves that worked by shrinking and expanding in response to temperature increments and decrements, respectively, allowing them to activate a specific channel using a heater [23]. 

c. Integration between the sensing device and detector. Considering wearable biosensors, another aspect that needs to be taken into account is the integration of the sensing element with a detector that should be implemented on the same platform. Generally, these biosensors are mainly based on electrochemical and optical detection since they can benefit from easy readout and data processing. Furthermore, thanks to the recent application of innovative nanomaterials technologies and the use of flexible and advanced platforms, they reached a sensitivity that allowed them to detect even small traces of the target analytes in complex matrices such as biofluid [3,24,25,26].

**Figure 1:**
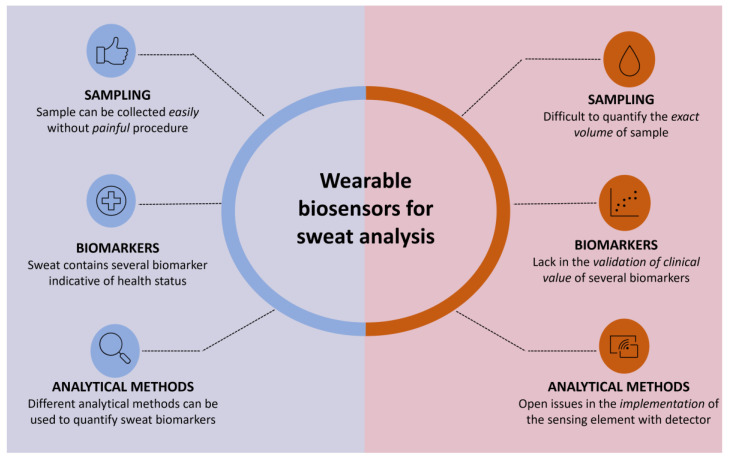
Advantages and open issues concerning the development of a biosensing system for sweat analysis.

Deeper understandings of sweat composition, together with advances in sampling and detection technologies, should accelerate the diffusion of sweat-based diagnostic devices for routine applications (Figure 1).

**Figure 2:**
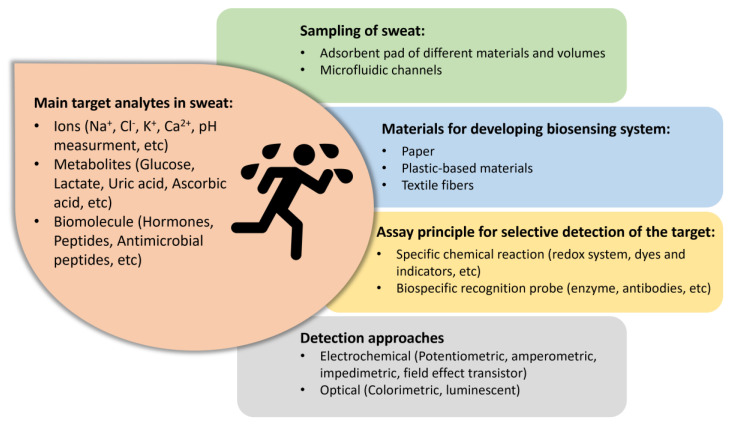
Issues in the development of wearable biosensors for sweat analysis.

This review aims to report the recent advances in skin-interfaced wearable biosensors for sweat analysis, which will pave the way for a non-invasive, personalized, and continuous monitoring system for a daily check of health status. Indeed, these systems can allow multiplex detection of several parameters, thus obtaining a more detailed clinical framework for a targeted diagnosis employing different analytical principles (enzymatic assay, immunoassay, or gene probe assay) combined with several detection strategies (mostly electrochemical and optical) and integrated with a wireless communication system for enabling POC monitoring (Figure 2). In particular, we will focus on the different microfluidic approaches that depend on the material selected for developing such biosensors (i.e., paper, highly flexible plastic, and textile). The review will critically highlight the open issues in obtaining a robust quantitative analysis, considering all the aspects involved in the analysis, including sampling, storing, and sensing capabilities. 

## 2. Paper-Based Microfluidic

Paper-based microfluidic devices (µPADs) applied to wearable biosensors result in particular advantages thanks to their low cost, high surface-area-to-volume ratio, non-toxicity, flexibility, and the possibility to combine sampling and storing of the reagents on the same platform [27]. Thanks to the capillary-based flow, it is also possible to move fluids across the device without external forces, making it suitable for power-free applications. μPADs are generally produced by employing chemical printing and/or cutting in order to obtain specific areas in which the biospecific recognition reactions take place. The microfluidic pattern can be represented by a 2D or 3D configuration, depending on the vertical or horizontal pathways selected for moving the fluids. This great variety of materials, procedures of treatment, and configurations pave the way for the development of different devices that can be designed ad hoc for the desired application [28,29,30,31].

Furthermore, several analytical paper-based devices for the quantification of biomarkers in sweat have been proposed in the literature [32,33,34], thus allowing a simple implementation on a wearable platform of the reported operating principle, detection approach, and sampling technique.

Concerning the wearable paper-based devices for sweat analysis, one of the major applications reported in the literature is glucose monitoring [35,36]. To date, however, most systems are aimed at multiparametric analysis, in which various analytes are quantified through a single analysis. Among the most reported multiplex applications, the simultaneous detection of glucose and lactate, together with pH measurement, is very widespread. The majority of these systems therefore integrate on the paper platform all the reagents necessary for the analysis, which in most cases include the specific enzymes for the recognition of the target analyte and their respective substrates, which allow the formation of a signal (generally colorimetric or electrochemical) to be acquired.

**Figure 3:**
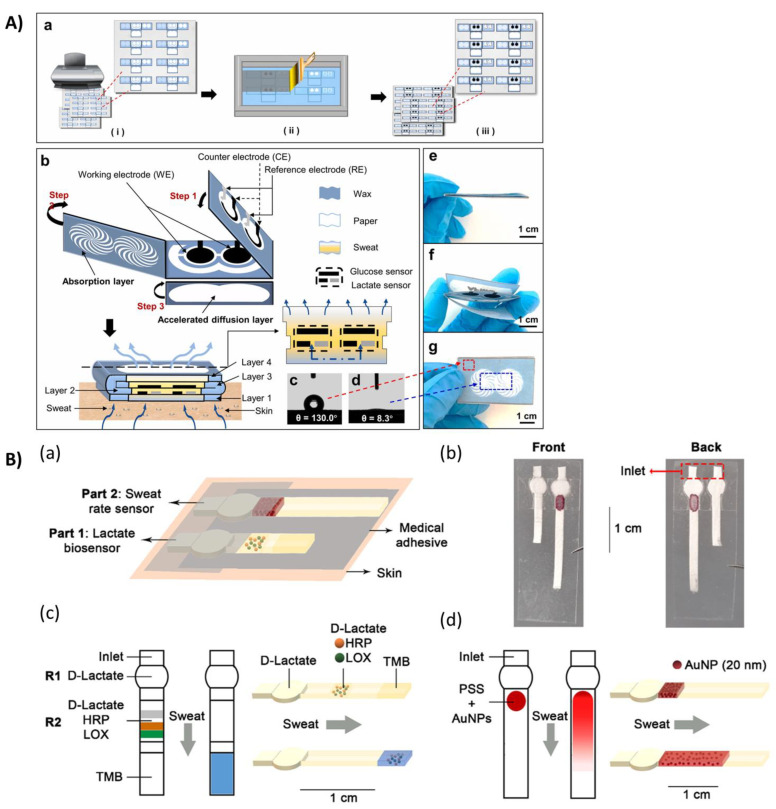
(**A**) Schematic illustration and photos of the highly integrated sensing (HIS) paper. (**a**) Schematic illustration of the fabrication of HIS paper. (**b**) Structural anatomy of HIS paper. (**c**,**d**) Contact angle tests on hydrophobic and hydrophilic regions of the HIS paper. (**e**,**g**) Photographs of the foldable HIS paper. Reprinted from Ref. [38] Copyright 2020 with permission from Elsevier; (**B**) Design of the wearable analytical platform. (**a**) Schematic representation and (**b**) photographs of the platform. Medical adhesive below the paper strips prevents contact with the skin, with the exception of the inlets. Another layer of medical adhesive above the strips presses the inlets against the skin. The area in contact with the skin is highlighted in red. (**c**) Lactate biosensor before and after sweat measurements. A circular area containing D-lactate is located right after the inlet (reservoir 1, R1). Another area containing D-lactate, horseradish peroxidase (HRP), and lactate oxidase (LOX) is located between the circular area and the detection zone (reservoir 2, R2). Finally, TMB is stored in the detection zone. (**d**) Sweat rate sensor before and after sweat measurements. A reservoir containing polystyrene sulfonate (PSS) and gold nanoparticles (AuNPs) is located right after the inlet. Reprinted with permission from Ref. [40] Copyright 2021 American Chemical Society.

Cao et al. [37] developed a wearable electrochemical biosensor based on different stacked paper layers obtained by wax screen printing technology that allowed for analysis by folding the layers sequentially. Since one of the major problems related to sweat analysis with wearable biosensors is the precision of the analyzed sample volume, the authors reported a simulation of the capillary flow across the proposed device, testing different amounts of volume and considering 5 s as the time required for the flow. Also, Li et al. [38] developed a foldable paper-based patch for detecting lactate and glucose simultaneously (Figure 3A). The sensing mechanism was based on electrochemical detection, exploiting Ti_3_C_2_T_x_ as a platform for immobilizing enzymes for the recognition reaction of the target analytes and as a substrate for the development of the analytical signals as well as a facilitator for charge migration. The authors also performed a detailed study about the diffusion path of sweat into the paper substrates in order to design a device that can allow an effective collection of sweat that is indicative of the concentration of the analytes of interest. 

Vaquer et al. [39] proposed a time-controlled sampling method for efficient monitoring through sequential measurements. In particular, they designed valves for analyzing a controlled volume of sweat by exploiting dried polymers that can be dissolved by the liquid, allowing the sample to achieve the functionalized area in which a specific enzyme (urease) was immobilized to react specifically with the target analyte (urea). The measurements could be performed in sequence thanks to the sequential valve opening in order to detect changes in the concentration of the analyte using the colorimetric approach. The same research group reported about a device in which they integrated a sweat volume sensor [40] (Figure 3B). This sensor consisted of a reservoir containing gold nanoparticles (AuNPs) that were carried out during the sampling step through a paper channel, and by measuring the distance covered by AuNPs, it was possible to evaluate the volume of the collected sweat. 

Another solution for sampling sweat was proposed by Yokus et al. [41]. They developed a hydrogel able to extract sweat from skin through the osmosis phenomenon, and they coupled it with a paper-based channel that exploited capillary forces to move the sample across the device. Furthermore, the evaporation of sweat from the paper pad enabled the presence of pressure inside the channel, which allowed the collection of the sample over time. The biosensor was used to measure lactate, exploiting an enzymatic approach and electrochemical detection. Liu et al. [42] collected sweat into a reservoir exploiting the capillary forces of Triton X-100-modified hydrophilic microchannels. The biosensor was composed of a hydrophilic PDMS layer integrated with microchannels and reservoirs, a PDMS cover layer, and a functionalized layer with the enzymes necessary for the recognition of the target analytes. The colorimetric signal for the quantification of glucose and the measurement of pH was acquired with a smartphone, and the images were elaborated to obtain a quantitative correlation. Cho et al. [43] described an enzymatic fuel cell composed of foldable paper in which the pattern for microfluidic and reservoir was obtained by wax printing. The electrochemical detection of glucose was obtained by exploiting a conducting polymer mixture (poly(3,4-ethylenedioxythiophene)/polystyrene sulfonate (PEDOT:PSS)) that acts both as the electroactive species and as a platform for immobilizing the enzyme. 

Another design comprising several layers was proposed by Zhang et al. [44]. The biosensing system comprised a top adhesive transparent layer for avoiding the evaporation of sweat, a wax-printed layer with a specific pattern for storing enzymes that have to react with the target analytes, an adhesive layer with some holes for enabling sweat inlet, and finally an easy-to-peel paper that has to be removed before attaching the chip to the skin. The readout of the colorimetric signal was performed with a smartphone for assessing the pH level and the quantity of glucose and lactate. Also, Liang et al. [45] reported about a foldable wax-printed patterned paper-based device composed of five layers: a sample collector, a vertical channel, a transverse channel, an electrode layer (comprising an potassium-selective screen-printed electrode), and a sweat evaporator. 

Gao et al. solved the problem of a controlled volume of sample by exploiting unidirectional sweat flow from the skin to the detection area of the biosensing system [46]. In particular, the fluid can move only in the direction of introduction from a hydrophobic to a hydrophilic area in which the reagent for the detection of target analytes has been immobilized. The device was able to measure pH, chloride, sodium, and glucose using a multiplex approach. 

Xiao et al. [47] reported about the possibility of exploiting hydrophobic silk thread as a biocompatible microchannel to make the sample flow toward the paper-based device functionalized with the enzyme for the colorimetric detection of lactate using a smartphone as a detector. The use of smartphones for quantifying color intensity in the context of colorimetric wearable biosensors is very widespread, but several issues have to be considered. Indeed, the acquisition of color signals can be affected by the ambient light and the difference in the equipment for taking the image, and these factors can lead to a problem in standardizing the analytical information. In this context, Shi et al. [48] proposed to include in the wearable analytical device a photosensor equipped with a LED as a light source for making uniform light during the acquisition of color signals. The data were then transmitted via wireless Bluetooth, and by exploiting a learning machine algorithm, it was possible to correlate the obtained data with the concentration of the target analyte. This approach made it possible to measure glucose, lactate, and pH. 

Cheng et al. [49] proposed a multiplex approach in which it was possible to detect several analytes simultaneously, exploiting both electrochemical and optical approaches. In particular, the device is based on the origami approach, and it combined different sensing strategies such as enzymatic reactions (for detecting glucose, lactate, and uric acid), pH indicators, complexes (for detecting magnesium ions), and molecularly imprinted polymers (for detecting cortisol). Wax printing technology was employed to create specific patterns for controlling the sequence of the specific reaction, and channels for delivering samples to the reactive areas were composed of thread. Electrochemical detection was used for the detection of cortisol, while other target analytes were detected through the formation of color. Another wearable biosensor for the detection of cortisol was proposed by Fiore et al. [50], who exploited an immunoassay combined with electrochemical detection. The authors optimized a competitive immunoassay based on the use of magnetic beads as a platform, and the results were transmitted to a smartphone by the near-field communication (NFC) wireless approach. A different application was reported by de Brito et al. [46]. The proposed biosensor was aimed at the detection of bacteria, *Staphylococcus aureus*, and it did not rely on the traditional protein-recognition assay. In particular, it exploited the phenomenon of the oxidation of ferrocyanide (produced upon the respiration cycle of the bacterium), and detection was performed electrochemically.

As it concerns detection approaches, the most commonly employed in the field of wearable paper-based biosensors are based on electrochemical and colorimetric strategies. Few examples have been reported exploiting different detection principles.

An example was reported by Ardalan et al. [51], who exploited fluorescence for detecting glucose, lactate, and chloride. Their paper-based biosensor was combined with a fluorescent imaging device. Cotton threads were used as microchannels to move fluids along the biosensing area on which reagents were immobilized. In particular, for glucose, the enzymatic reaction involving glucose oxidase was exploited in order to produce hydrogen peroxide able to oxidize fluorescein, causing the emission at a specific wavelength, while for lactate and chloride, a mechanism based on quenching of fluorescence was exploited. Another approach was selected by Mogera et al. [52].

Since the enzymes and antibodies can be affected by degradation and are characterized by a limited shelf life, the authors proposed the use of label-free surface-enhanced Raman spectroscopy (SERS) for quantifying uric acid in sweat. A serpentine channel was designed for allowing the flow of the excreted sweat through the plasmonic sensors immobilized at different areas of the paper substrate, and the detection was performed using a portable Raman spectrometer with a flexible fiber probe for spectra collection.

Paper-based systems are ideal for wearable applications thanks to paper’s intrinsic nature of flexibility and biocompatibility. Many examples in the literature are based on this platform, focusing more on analytes that can be detected through enzymatic biospecific recognition reactions. Detection systems generally rely on electrochemical and colorimetric methods, which are very suitable to be implemented on wearable biosensors. A resume of the reported work based on paper-wearable biosensing systems is reported in Table 1. 

Indeed, the formation of color on the surface of the paper is clearly visible to the naked eye, and the intensity of the color can be quantified using portable and easy-to-use instruments such as the smartphone camera. Similarly, electrochemical detection can be carried out using portable potentiostats, or recently, the signal can also be transmitted to the smartphone using NFC wireless technologies. For the moment, therefore, the trend is to adapt enzymatic paper-based biosensors, which are already widely used for routine applications, to wearable platforms. Probably in the future, once these systems are optimized starting from standardized and well-known methods, other biosensing approaches and detection techniques will also be evaluated for their integration into wearable platforms.

## 3. Wearable Biosensors Based on Highly Flexible Plastic Substrate

Among the flexible and stretchable materials for developing wearable biosensors, polymers like polyethylene terephthalate (PET), polyimide (PI), polydimethylsiloxane (PDMS), polyurethane (PU), and polymethyl methacrylate (PMMA) are very widespread. Indeed, thanks to their low cost, biocompatibility, physical-mechanical properties, and electrical insulation, they are optimal candidates as substrates for biosensing devices [54,55]. Furthermore, several portable biosensors have already been developed exploiting such materials, allowing for many examples of the functionalization of these materials with biomolecules, leading to the easy implementation of these biosensing systems on a wearable platform. 

Gao et al. [56] presented a wearable biosensor for simultaneous detection of a panel of biomarkers in sweat. The sensor was produced on a PET substrate, while flexible printed circuit board (FPCB) technology was used to implement the signal processing and exploit integrated-circuit components. As it concerns the target analytes, glucose and lactate were detected using glucose and lactate oxidase immobilized on a film of polysaccharide chitosan. For the measurement of Na*^+^* and K*^+^*, an ion-selective electrode coupled with a polyvinyl butyral (PVB)-coated reference electrode was used. 

Park et al. [57] reported about the use of a polyimide substrate integrated onto a needle to be used during medical procedures, on which were electrodeposited reactive layers for the detection of glucose and lactate. Six microelectrodes were deposited on a polyamide membrane, and then on each electrode, enzymes were immobilized for detecting the target analytes as well as metal oxide for the measurement of the pH. 

**Figure 4:**
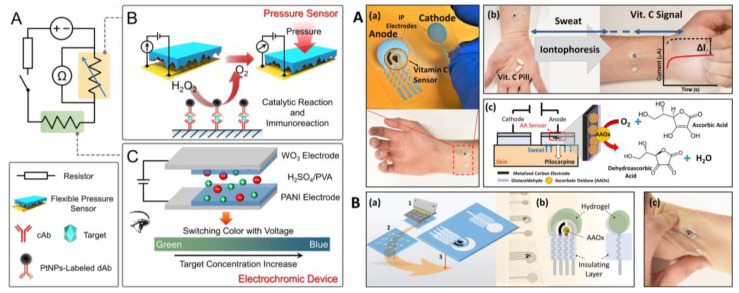
(**Left**) Scheme of the electrochromic device. (**A**) Circuit diagram of the pressure-based immunoassay platform; (**B**) schematic illustration of the PtNP-triggered immunoassay with a flexible pressure sensor readout; and (**C**) structure of the voltage-regulated electrochromic device as a visualized readout. Reprinted with permission from [59]. Copyright 2021 American Chemical Society. (**Right**) Ascorbic acid (vitamin C) determination in stimulated sweat. (**A**) (**a**) Electrode design for simultaneous sweat stimulation and detection. Sweat is stimulated by IP delivery of pilocarpine (located in the anode compartment) using the cathode and anode electrodes. Amperometric vitamin C detection is performed by using a three-electrode system located in the anode compartment. (**b**) Protocol used for the biosensing of ascorbic acid. Sweat is stimulated before (black dotted line) and after (red solid line) taking vitamin C pills; the vitamin C response is based on the difference in the current before and after taking the pill. (**c**) Schematic of the localized sweat stimulation using IP pilocarpine delivery and of the enzymatic reaction for detecting ascorbic acid on a metalized Rh-carbon printed electrode. Pilocarpine is delivered on the anode, where the AAOx-immobilized sensor using glutaraldehyde is located; the amount of oxygen consumed by the enzymatic reaction and hence the vitamin concentration are measured from the reduction current of oxygen. (**B**) (**a**) Fabrication of the vitamin C biosensor. 1—Screen printing using Ag/AgCl for the IP electrodes, reference and current collectors, and Rh-carbon ink for the counter and working electrodes. 2—printed and cured electrodes. 3—printing an insulating layer to define the electrode area. (**b**) Schematic showing the location of the hydrogel and the enzyme layer. On the anode, agarose (loaded with 2% pilocarpine) was used, and on the cathode, agarose in 0.1 M PBS was used. (**c**) Image of the epidermal sensor under mechanical (twisting) strain. Reprinted with permission from [60] Copyright 2020 American Chemical Society.

Xu et al. [58] proposed an electrochemical wearable sensing system in which electrodes were printed on PDMS, exploiting conductive silver wires for the connections. Using NFC technology, data were transmitted to smartphones for detecting glucose, sodium, potassium, and pH values. Thanks to the design of the biosensor, there was no need for a battery or wires. Sampling of sweat was performed by using a porous sponge placed on the electrodes, allowing for the creation of a liquid environment in which the electrodes could work properly. A different mechanism was presented by Yu et al. [59], who proposed a pressure-based biosensor for the detection of carcinoembryonic antigen (Figure 4 left). A non-competitive immunoassay was optimized, in which the detection antibody was functionalized with platinum nanoparticles (PtNPs) as a tracer. The recognition of the target analytes caused a pressure signal thanks to the decomposition of hydrogen peroxide into O*_2_*. When the pressure increases, the biosensor undergoes elastic deformation, leading to a change in resistance and consequently to the generation of an electric signal. Furthermore, the applied voltage allowed a change in color, so the device can also be used for rapid detection using the naked eye. 

Another example was reported by Sempionatto et al. [60], who proposed an electrochemical sensor able to quantify vitamin C in sweat (Figure 4 right). They immobilized ascorbate oxidase on screen-printed electrodes (SPE), which were deposited on a polyurethane substrate. The enzyme is able to catalyze the oxidation of vitamin C, leading to the consumption of oxygen, which was monitored by measuring the reduction current. The device was implemented with a localized pilocarpine-based iontophoretic sweat stimulation system in order to improve the sampling step. Also, Imani et al. [61] designed a wearable device in which the working electrode was deposited on a flexible polyester platform suitable for adhering to the human skin. They were able to quantify lactate by exploiting an enzymatic assay in which lactate oxidase coated the working electrode that was printed using Prussian blue ink, which is suitable for measuring the hydrogen peroxide developed from the enzymatic reaction between lactate and lactate oxidase. Since the biosensor was also used for the physical determination of electrophysiological parameters, a hydrophobic layer was used to separate the electrode for measuring lactate from those for the acquisition of electrocardiograms. 

The same research group also proposed several works based on the tattoo approach [62,63,64,65,66]. In particular, a tattoo-based biosensor for non-invasive lactate monitoring is based on an amperometric sensor [67,68]. The working electrode was prepared following the SPE technique on a biocompatible substrate of chitosan, and it was functionalized with tetrathiafulvalene (TTF), multi-walled carbon nanotubes (CNT), and lactate oxidase. 

Unlike paper-based wearable biosensors, which are very suitable for developing analytical devices based on colorimetric detection, those made of plastic materials are mostly coupled with electrochemical detection. One of the rare examples was reported by Curto et al. [69]. The authors developed a disposable micro-fluidic platform exploiting ionogels for monitoring in real time the pH of sweat during an exercise through a barcode color variation when compared to a standard color chart. The detection can be performed with the naked eye or also by coupling simple opto-electronic components integrable into the device in order to obtain a continuous feedback of the sweat pH. 

The microfluidic platform was composed of four reservoirs and channels, fabricated in six layers of poly(methyl methacrylate) and PSA, with four ionogels containing pH-sensitive dyes (methyl red, bromocresol green, bromocresol purple, and bromothymol blue). The microchannels connected the reservoir filled with ionogel and dyes with a bigger reservoir, where an absorbent fiber drives the fresh sweat by capillary action from the skin to the sensing area. Another optical approach was exploited by Sekine et al. [70]. They exploited fluorescent detection using smartphones combined with imaging accessories for determining chloride, sodium, and zinc. Target analytes reacted in microreservoirs containing fluorescent probes that, upon reaction, were able to change the intensity of fluorescence. The analytical device, made principally of PDMS and PMMA, comprised a skin-compatible adhesive layer, a platform of microfluidic channels and valve structures that drive sweat to microreservoirs containing fluorescent probes, and a light-shielding layer to prevent exposure of these reagents to light before the measurements. As it concerns the detection module, a dark shielded box with excitation and emission filters allowed the capture of fluorescent images using a smartphone camera and exploiting the LED flash as a source of excitation light. 

The development of wearable biosensors based on plastic materials is very advantageous from the point of view of the great variety of substrates available with different properties depending on the selected material. The high versatility offered by the plastic-based substrates, however, does not solve the problem of the mismatch between these materials and the skin, which leads to an increase in sweat production and its consequent accumulation, making the quantitative test distorted. Even if the breathability of these devices has improved significantly in the last few years, some steps should be taken to make them usable over a long period of time. 

## 4. Textile Materials for Developing Sweat-Real-Time Monitoring Biosensors

The intrinsic properties of textile materials make them ideal candidates for the development of wearable biosensors, enhancing the chances of success for these devices. Indeed, biocompatibility, softness, lightweightness, permeability, and breathability can be exploited in order to obtain a biosensor that can be kept in contact with the skin, avoiding wearable discomfort [71,72].

Among the advantages of exploiting textile materials is the possibility of improving sweat collection, which remains one of the major challenges in the field of wearable biosensors. Indeed, the transport of absorbed liquids through the hydrophilic cotton thread was also used for developing wearable biosensors based on paper materials, since it is an approach that combines biocompatibility with a directional liquid transport ability. Several textile materials are available for developing sensing and analytical devices. Among them, natural materials such as cellulose, silk, chitosan, etc. are the most exploited. Cellulose can be employed in different structures (e.g., fabric, yarn, etc.), and it is ideal for collecting and transporting sweat thanks to its high wiking rate [73,74]. Differently from cellulose, silk fibroin is generally used for the immobilization of enzymes since its structure allows it to maintain stability over time [75,76,77]. Moreover, carbonized silk combines high electrical conductivity with improved electron transfer ability [77].

As for paper and plastic materials, textile wearable biosensors are also mostly based on electrochemical methods, while fewer examples were reported of optical detection. 

Khan et al. [78] developed a cotton-based biosensor for monitoring lactate in sweat by employing a three-electrode system. In this configuration, lactate oxidase dispersed in a chitosan solution was deposited on the working electrode, which was chemically modified with a graphite-polyurethane-reduced graphene oxide (G-PU-RGO-PB) paste. Zhao et al. [79] proposed fiber-based triboelectric nanogenerators produced by covering multiwalled carbon nanotubes (MWCNTs) and polyaniline on an ecoflex fiber. The device was powered by coupling the triboelectric effect with an enzymatic reaction, and it allowed the detection of glucose, creatinine, and lactate acid in real-time.

Silk-derived nitrogen-doped carbon textile (SilkNCT) was employed by He et al. [80] for developing a multiplex biosensing system for the detection of glucose, lactate, ascorbic acid, uric acid, Na^+^, and K^+^. Six electrochemical sensors are integrated into the device, with SilkNCT acting as the working electrode. The circuit was produced using a digital laser writing technique on conductive Ni-coated textile tape. Also, Chen et al. [77] exploited silk fibers of reduced graphene oxide (RGO) functionalized with glucose oxidase to obtain a working electrode for developing sweat glucose sensing devices.

In addition to the traditional potentiometric, amperometric, and impedimetric approaches, one of the most successful strategies in the field of textile biosensing is the field-effect transistor. In particular, organic electrochemical transistors (OECTs) are composed of a strip of conductive polymer (e.g., poly(3,4-ethylenedioxythiophene, PEDOT) that acts as a channel and an electrode, separated by an electrolyte solution. Electrochemical reactions can modulate the current and the conductivity of the channel, thus making OECT amplifiers of the electrochemical signal. OECTs can be easily embedded in textile materials because they do not need the traditional design consisting of three electrodes, making the necessary equipment simpler than the potentiostats that are generally employed for electrochemical sensors [81,82].

In this context, Possanzini et al. [83] proposed a textile sensor based on natural and synthetic fibers, coated with the conducting polymer poly(3,4-ethylenedioxythiophene)/poly(styrene-sulfonate) (PEDOT:PSS), and suitably functionalized with a nano-composite material or a chemical dye to measure Cl^−^ and pH. The same research group also developed a bendage for monitoring the wound pH in real time by integrating a sensing system based on a pH sensor made of a semiconducting polymer PEDOT:PSS thin film comprising embedded iridium oxide particles with an adsorbent layer for delivering wound exudate to the sensing area [84].

**Figure 5:**
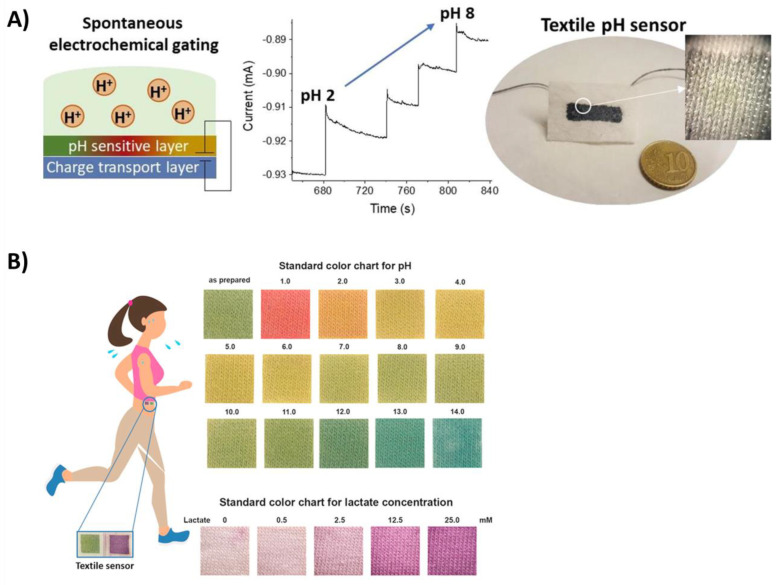
(**A**) Scheme of the pH sensing system with the two-terminal device. Reprinted from Ref. [85]. Copyright (2018), with permission from Elsevier. (**B**) pH values between 0 and 14 and lactate concentrations between 0.5 and 25 mM could be distinguished by the color changes. Reprinted from Ref. [90] Copyright (2018), with permission from Elsevier.

Another example reported by Mariani et al. [85] for the measurement of pH was based on a PEDOT:PSS layer combined with a pH-sensitive layer, which was used as the gate material for PEDOT doped with Bromothymol Blue (PEDOT:BTB) (Figure 5A). The same approach based on the use of PEDOT:PSS was employed by Coppedè et al. [86], who functionalized this platform with an ion-selective membrane based on different ionophores in order to measure electrolytes in sweat. 

Concerning the colorimetric detection applied to wearable biosensors based on textile materials, most of the reported works are aimed at pH evaluation. Indeed, by detecting the color change with the naked eye or using a spectrophotometer, it is possible to monitor sweat pH in real time. pH-sensitive dyes and halochromic materials whose colors depend on the pH change can be easily embedded into textiles. 

Caldara et al. [87] employed litmus, which is a halochromic dye, in order to obtain a hybrid sol-gel matrix for soaking cotton-based material and develop a system in which the color shifted from red to blue depending on the pH of the sweat. For a better evaluation of the color and a more precise measurement of the pH, the authors combined the proposed system with an optoelectronic circuit placed in front of the device, which worked in a reflective way. The device was enclosed in a dark box, integrating a white LED to maintain homogeneous illumination and an RGB photodiode to collect the reflected light. Also, Morris et al. [88] integrated the colorimetric pH detection system into a portable detector based on LEDs to measure color changes reproducibly. The authors focused on the development of a self-standing system comprising a sensitive pH dye integrated into the textile material and a fluid handling platform based on a passive pump for collecting sweat and moving it through channels. Yapor et al. [89] developed nanofibers that were composed of 10,12-pentacosadiynoic acid (PCDA) mixed with a supporting polymer including poly(ethylene oxide) (PEO) and polyurethane (PU) and showed colorimetric responses to external stimuli such as the presence of E. coli and changes in pH. Promphet et al. [90] obtained a wearable biosensor for measurement of pH and lactate by depositing three layers on a cotton fabric (Figure 5B). The layers included chitosan, sodium carboxymethyl cellulose, and an indicator dye (a mixture of methyl orange and bromocresol green) or lactate oxidase. By comparing the obtained colors with a standard calibration, the proposed system was able to estimate the sweat pH (1–14) and the lactate concentration (0–25 mM). Zhao et al. [91] developed a biosensing platform composed of thread-embroidered patterns that, after treatment with the chromogenic reagents for performing the bioassays, can be used as detection areas in which sweat is transferred thanks to hydrophilic threads. The system allowed for the evaluation of local sweat loss, pH, chloride, and glucose concentrations by exploiting a colorimetric reference standard or by acquiring digital images obtained with a smartphone’s camera and elaborated using prestored calibration curves. 

Researchers have made great efforts to develop biosensing systems exploiting textile materials that can be biocompatible and more comfortable than plastic or paper-based substrates. With respect to the first prototypes, the trend is to exploit natural sustainable fibers to replace synthetic materials, which can have uncomfortable reactions for long-term contact with the skin as well as poor degradation and environmental friendliness. Among the issues remaining unsolved is the need for frequent calibration processes and the use of electrodes, which should combine low-cost process preparation, good stability, and performance. Indeed, sweat biosensors based on textile materials tend to lose reproducibility after long-term use due to contamination and damage. On the other side, the colorimetric approach is generally applied in a disposable context, which is not sustainable for large-scale use. 

## 5. Outlook and Perspectives

**Figure 6:**
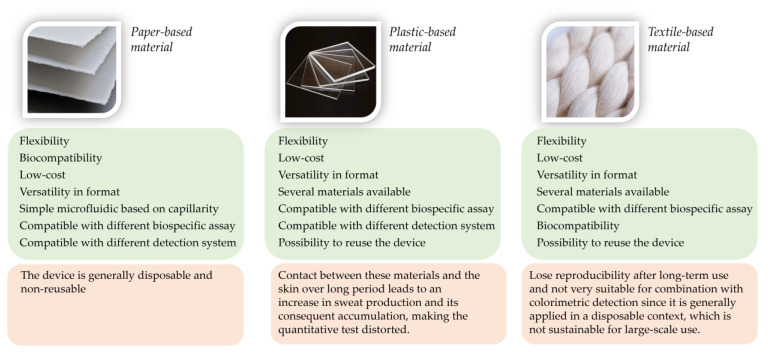
Scheme of pros and cons of exploiting different formats for developing wearable biosensing systems.

The development of wearable technologies has attracted much attention in the last few years, emerging as a promising approach for the clinical diagnostic field in the near future. Indeed, the possibility of obtaining information about target analytes in real time, non-invasively, and outside of centralized laboratories represents a very interesting approach that could have positive implications in everyday life. Several biosensors have been proposed in the literature based on different platforms, materials, and detection approaches. Figure 6 shows a summary of the main advantages and disadvantages of the innovative materials selected for the development of these devices. Despite the great efforts and innovations made in this field, wearable biosensors for sweat analysis are not yet commercially widespread. This fact derives mainly from some points that still remain open issues. First of all, there is the possibility of using more complex analytical systems compared to the use of pH indicators or the enzymatic measurement of some target analytes. Indeed, the most widespread systems are those based on the integration within the device of specific reagents (indicators, dyes, etc.) or enzymes for the measurement mainly of pH, electrolytes, glucose, and lactate, but the implementation of more complex immunological assays or bioassays is still behind. The possibility of exploiting more complex recognition mechanisms would lead to an increase in detectable target analytes (including hormones, proteins, and peptides) through these platforms that can be of broader interest. In this context, the validation of biomarkers and their concentrations in physiological or indicative situations of a pathology would certainly be very useful in order to be able to develop devices characterized by high diagnostic utility. For achieving this goal, the development of sweat-wearable biosensors should be combined with a big data approach, including statistical analysis, pattern recognition, and the use of artificial intelligence for obtaining correlations and accurate predictions [92,93]. Indeed, correlation studies can identify the profiles of analytes of interest that can maximize the amount of information obtainable from a single analysis. The application of big data resources has already proven to be very crucial for diagnostic screening, providing early warnings for several pathologies. This approach would also favor greater compliance with the goal of personalized medicine, which implies a diagnostic pattern focused on individual health status. 

Another point that needs further progress is the collection of a reproducible and precise volume of the sweat sample, which is essential to performing the correct quantification of the target analyte. Microfluidics is essential for solving this open issue, and recently some designs were proposed. The multi-material approach guarantees good control of the volume collected as well as high flexibility of the device. The selection of materials and design for a wearable biosensing system should take into account the combination of biocompatibility, low cost, and good mechanical properties. For achieving these goals, 3D printing technologies represent irreplaceable tools, as nowadays they are able to exploit several innovative inexpensive materials (i.e., hydrogels, ionogels, elastomers, etc.) that allow to produce microfluidic systems characterized by high resolution as well as rapidity of fabrication. As regards detection systems, the electrochemical one, which allows real-time monitoring of the concentration of target analytes, is the most widespread. Optical detection methods are also of great interest, especially in the paper-based format, even if the use of irreversible colorimetric reactions makes them less suitable for the creation of biosensors that should be reusable and sustainable from an environmental point of view [94]. In any case, the performances offered by the different approaches for detection are comparable, as shown in Table 2, which reports a review of a wearable biosensing system developed for glucose detection in sweat. 

Finally, the implementation of these wearable systems with highly sensitive detectors represents a further step to be considered in view of their use in everyday life. Indeed, integration with detection systems that allow greater reliability of the data obtained is one of the points on which it is necessary to find solutions that combine high sensitivity with miniaturization of the device and simplicity of the interface for the acquisition and processing of the obtained data.

## Figures and Tables

**Table 1:** Paper-based wearable biosensors for sweat biomarker quantification.

Target Analyte	Detection Principle	Limit of Detection	Ref
Glucose	Wax screen-printing patterns on cellulose paper are folded to form five stacked layers: the sweat collector, vertical channel, transverse channel, electrode layer, and sweat evaporator. A screen-printed glucose sensor based on a glucose oxidase enzymatic reaction was developed on a polyethylene terephthalate substrate.	5 μM	[37]
Glucose and lactate	Printed paper folded into a multi-layer structure was designed to incorporate into the same platform conducting electrodes, specific enzymes, and MXene/methylene blue. The sweat diffusion path was established by connecting the hydrophilic regions of each layer along the vertical direction of the folded paper.	Glucose 17.05 μM Lactate 3.73 μM	[38]
pH and urea	Dried polymers were used as closed valves that deflect the flow of liquids to different transducers in a multisensor. As time passes, the polymer dissolves, and the valve opens. The sequential opening of the valves results in a succession of measurements that reveal fluctuations in the concentration of the target analyte. The colorimetric detection was performed using dyes for pH detection and urease for the enzymatic reaction involving urea.	Urea 5 mM	[39]
Lactate	The detection platform consists of two adjacent sensors (one for detecting lactate and one related to the sweat volume) made entirely of paper whose inlets are in direct contact with the skin. Lactate was detected through the colorimetric reaction involving lactate oxidase/HRP/TMB.	0.06 mM	[40]
Lactate	The patch consisted of a hydrogel and paper-based microfluidic device for combined osmotic and capillary pumping of sweat, coupled with screen-printed electrodes for analysis of lactate concentration by exploiting an enzymatic reaction based on lactate oxidase.	2 mM	[41]
Glucose and pH	A PDMS-based chip was modified with nonionic surfactants for the automatic collection of sweat combined with a paper-based sensor based on colorimetric measurement of pH and enzymatic reaction for detecting glucose using a smartphone for image acquisition.	0.05 mM	[42]
Glucose	The paper-based device wicks sweat by using capillary forces in a reservoir where glucose is detected electrochemically by exploiting catalytic reactions. The device used a 3D paper-based fuel cell configuration, an electrically conducting microfluidic reservoir for a high anode surface area and efficient mass transfer, and a direct electron transfer between glucose oxidase and anodes for enhanced electron discharge properties.	0.02 mg/mL	[43]
pH, glucose, and lactate	The channels/patterns were designed and printed on the paper using a wax printer. These channels directed the sweat to the collection and detection zones of the device, where the intake, storage, and evaporation of sweat were designed to maximize the efficiency of the colorimetric catalytic reactions. The measurement was performed using a smartphone.	Glucose 50 μM Lactate 5 mM	[44]
Potassium	The paper-based microfluidic pad was fabricated by printing wax patterns on cellulose paper and then folding the pre-patterned paper to form a five-layer stacked structure: sweat collector, vertical channel, transverse channel, electrode layer, and sweat evaporator.	1 mM	[45]
pH, chloride, sodium, and glucose	A single sheet of paper was double-sided wax-printed and heat-treated for asymmetric wettability, allowing unidirectional sweat transport and delivering the sweat to the sensing areas for multiplexed colorimetric assays.	Chloride 20 mM Sodium 40 mM Glucose 0.1 mM	[46]
Lactate, pH	Hydrophilic silk thread serves as the micro-channel to guide the liquid flow, while filter papers were functionalized to prepare colorimetric sensors for lactate and pH measurements performed by smartphone cameras.	0.98 mM	[47]
Lactate, glucose, and pH	A paper-based analytical sensor was integrated into a PDMS microfluidic chamber prepared through 3D printing. A photoelectric sensor with an LED light source was used for acquiring the color information (including R, G, B, Lux, and color temperature value), and the data were transmitted wirelessly with Bluetooth.	Glucose 50 μM Lactate 10 mM	[48]
Glucose, lactate, uric acid, magnesium ions, and pH (colorimetric detection) Cortisol (electrochemical detection)	The entire chip is composed of hydrophilically and hydrophobically treated filter paper, and 3D microfluidic channels are constructed by using folding paper. The thread-based channels formed after the hydrophilic and hydrophobic modifications are used to control the rate of sweat flow, which in turn can be used to control the sequence of reactions in the differently developing colored regions to ensure that signals of the best color can be captured simultaneously by a smartphone camera. Cortisol was detected electrochemically using molecular imprinting polymers as probes for the recognition reaction.	Glucose 10 μM Lactate 2 mM Uric acid 2 μM Magnesium ion 0.5 mM Cortisol 100 ppm	[49]
Cortisol	The paper-based microfluidic pattern was made using wax printing and laser-cutter techniques for the delivery of capillary-driven microfluidics. The presence of magnetic beads functionalized with monoclonal antibodies for the recognition of the cortisol in the reaction zone allows a competitive reaction between the target cortisol and the labeled cortisol with the acetylcholinesterase enzyme, giving an electrochemical response by simply folding the pad loaded with the enzymatic substrate.	10 ng/mL	[50]
*Staphylococcus aureus* through the detection of K_4_Fe[CN]_6_	Paper-derived carbon electrodes were modified with a thin layer of sputtered gold and with chitosan. The resulting material was laser-patterned and applied for the development of an electrochemical biosensor wirelessly controlled by a custom-built, portable potentiostat.	8.6 μM	[51]
Glucose, lactate, pH, and chloride	The sensor comprised fluorescent sensing probes embedded in paper substrates, and microfluidic channels consisted of cotton threads to harvest sweat from the skin surface and transport it to the paper-based sensing probes. The imaging module was fabricated by a 3D printer equipped with UV-LED lamps and an optical filter to provide the in situ capability of capturing digital images of the sensors via a smartphone.	Glucose 7 μM Lactate 0.4 mM Chloride 5 mM	[52]
Uric acid	Plasmonic paper-based microfluidics based on label-free surface-enhanced Raman spectroscopy (SERS) for providing “fingerprint” information for analyte identification.	20 μM	[53]

**Table 2:** Wearable biosensing devices for the detection of glucose in sweat.

Platform	Detection Principle	Limit of Detection	Ref
Paper-based	Electrochemical	5 µM	[37]
Paper-based	Electrochemical	17.05 μM	[38]
Paper-based	Colorimetric	0.05 mM	[42]
Paper-based	Electrochemical	0.02 mg/mL	[43]
Paper-based	Colorimetric	50 µM	[44]
Paper-based	Colorimetric	50 µM	[48]
Paper based	Fluorescent	10 µM	[52]
PET	Electrochemical	50 µM	[56]
Polyamide	Electrochemical	0.5 mM	[57]
PDMS	Electrochemical	100 µM	[58]
Ecoflex fiber	Electrochemical	0.0056 mmol/L	[79]
SilkNCT	Electrochemical	5 µM	[80]
SilkRGO	Electrochemical	300 nM	[77]
Cotton and regenerated cellulose yarn	Colorimetric	10 μM	[91]

## Data Availability

Not applicable.
